# Supplementary material for: IER5, a DNA damage response gene, is required for Notch-mediated induction of squamous cell differentiation
Source: eLife. 2020 Sep 16;9:e58081. doi: 10.7554/eLife.58081 (PMC7529455; doi:10.7554/eLife.58081)
Supplement: Transparent reporting form [file elife-58081-transrepform.dotx]

***eLife’s* transparent reporting form**

We encourage authors to provide detailed information *within their submission* to facilitate the interpretation and replication of experiments. Authors can upload supporting documentation to indicate the use of appropriate reporting guidelines for health-related research (see [EQUATOR Network](http://www.equator-network.org/%20)), life science research (see the [BioSharing Information Resource](https://biosharing.org/)), or the [ARRIVE guidelines](http://www.plosbiology.org/article/info:doi/10.1371/journal.pbio.1000412) for reporting work involving animal research. Where applicable, authors should refer to any relevant reporting standards documents in this form.

If you have any questions, please consult our Journal Policies and/or contact us: [editorial@elifesciences.org](mailto:editorial@elifesciences.org).

**Sample-size estimation**

- You should state whether an appropriate sample size was computed when the study was being designed
- You should state the statistical method of sample size computation and any required assumptions
- If no explicit power analysis was used, you should describe how you decided what sample (replicate) size (number) to use

Please outline where this information can be found within the submission (e.g., sections or figure legends), or explain why this information doesn’t apply to your submission:

As is standard in the field, all RNA expression studies were designed to include at a minimum biological replicates, and Notch-dependent differential expression of genes of interest was confirmed by focused RT-PCR analysis and/or by Western blotting in independent experiments.

**Replicates**

- You should report how often each experiment was performed
- You should include a definition of biological versus technical replication
- The data obtained should be provided and sufficient information should be provided to indicate the number of independent biological and/or technical replicates
- If you encountered any outliers, you should describe how these were handled
- Criteria for exclusion/inclusion of data should be clearly stated
- High-throughput sequence data should be uploaded before submission, with a private link for reviewers provided (these are available from both GEO and ArrayExpress)

Please outline where this information can be found within the submission (e.g., sections or figure legends), or explain why this information doesn’t apply to your submission:

Our definition of biological replicates is standard in the field; these are parallel measurements of biologically distinct samples. Numbers of independent biological replicates performed are stated in the legends to each figure. All data points were included in the analyses presented. RNA-seq and ChIP-Seq data sets for SC2 cells in the Notch-on and -off states as well as RNA-seq data sets for control SC2 cells, SC2 cells knocked out for IER5, and SC2 cells with IER5 expression restored are available at https://www.ncbi.nlm.nih.gov/geo/query/acc.cgi?acc=GSE156488. RNA-seq data sets for IC8 cells treated with vehicle or GSI are available at <https://www.ncbi.nlm.nih.gov/geo/query/acc.cgi?acc=GSE156624>. All protein-based analyses (Western blotting, immunohistochemistry, immunofluorescence) were repeated at least one time in an independent experiment. RNA-seq, ChIP-Seq, and mass spectroscopic experiments are expensive, and we did not replicate experiments that produced results that passed quality control metrics. However, all key findings have been confirmed in orthogonal studies (e.g., reporter gene assays, RT-PCR assays, Westerin blotting, immunohistochemistriy, immunoprecipitation studies, and binding assays in purified systems).

**Statistical reporting**

- Statistical analysis methods should be described and justified
- Raw data should be presented in figures whenever informative to do so (typically when N per group is less than 10)
- For each experiment, you should identify the statistical tests used, exact values of N, definitions of center, methods of multiple test correction, and dispersion and precision measures (e.g., mean, median, SD, SEM, confidence intervals; and, for the major substantive results, a measure of effect size (e.g., Pearson's r, Cohen's d)
- Report exact p-values wherever possible alongside the summary statistics and 95% confidence intervals. These should be reported for all key questions and not only when the p-value is less than 0.05.

Please outline where this information can be found within the submission (e.g., sections or figure legends), or explain why this information doesn’t apply to your submission:

The Materials and Methods section provides detailed information describing the processing and analysis of targeted exonic DNA sequences, ChIP-seq data sets, and RNA-seq data sets using up-to-date informatics tools. Statistical analyses of RT-PCR data were conducted using the two-tailed student t test. All data points presented represent the means of replicates +/- SEM or +/- SD, depending on the analysis in question. These details can be found in the figure legends.

(For large datasets, or papers with a very large number of statistical tests, you may upload a single table file with tests, Ns, etc., with reference to sections in the manuscript.)

**Group allocation**

- Indicate how samples were allocated into experimental groups (in the case of clinical studies, please specify allocation to treatment method); if randomization was used, please also state if restricted randomization was applied
- Indicate if masking was used during group allocation, data collection and/or data analysis

Please outline where this information can be found within the submission (e.g., sections or figure legends), or explain why this information doesn’t apply to your submission:

Not applicable

**Additional data files (“source data”)**

- We encourage you to upload relevant additional data files, such as numerical data that are represented as a graph in a figure, or as a summary table
- Where provided, these should be in the most useful format, and they can be uploaded as “Source data” files linked to a main figure or table
- Include model definition files including the full list of parameters used
- Include code used for data analysis (e.g., R, MatLab)
- Avoid stating that data files are “available upon request”

Please indicate the figures or tables for which source data files have been provided:

Not applicable
